# Exposure to ultrafine carbon particles at levels below detectable pulmonary inflammation affects cardiovascular performance in spontaneously hypertensive rats

**DOI:** 10.1186/1743-8977-5-19

**Published:** 2008-12-04

**Authors:** Swapna Upadhyay, Tobias Stoeger, Volkar Harder, Ronald F Thomas, Mette C Schladweiler, Manuela Semmler-Behnke, Shinji Takenaka, Erwin Karg, Peter Reitmeir, Michael Bader, Andreas Stampfl, Urmila P Kodavanti, Holger Schulz

**Affiliations:** 1 Institute for Inhalation Biology, HelmholtzZentrum München, German Research Center for Environmental Health, Neuherberg, Germany; 2 EPA-NHEERL, Research Triangle Park, NC 27709, USA; 3 Institute of Health Economics and Health Care Management, HelmholtzZentrum München, German Research Center for Environmental Health, Neuherberg, Germany; 4 Molecular Biology of Peptide Hormones, Max Delbrück Center for Molecular Medicine, Berlin, Germany; 5 Institute of Toxicology, HelmholtzZentrum München, German Research Center for Environmental Health, Neuherberg, Germany

## Abstract

**Background:**

Exposure to particulate matter is a risk factor for cardiopulmonary disease but the underlying molecular mechanisms remain poorly understood. In the present study we sought to investigate the cardiopulmonary responses on spontaneously hypertensive rats (SHRs) following inhalation of UfCPs (24 h, 172 μg·m^-3^), to assess whether compromised animals (SHR) exhibit a different response pattern compared to the previously studied healthy rats (WKY).

**Methods:**

Cardiophysiological response in SHRs was analyzed using radiotelemetry. Blood pressure (BP) and its biomarkers plasma renin-angiotensin system were also assessed. Lung and cardiac mRNA expressions for markers of oxidative stress (hemeoxygenase-1), blood coagulation (tissue factor, plasminogen activator inhibitor-1), and endothelial function (endothelin-1, and endothelin receptors A and B) were analyzed following UfCPs exposure in SHRs. UfCPs-mediated inflammatory responses were assessed from broncho-alveolar-lavage fluid (BALF).

**Results:**

Increased BP and heart rate (HR) by about 5% with a lag of 1–3 days were detected in UfCPs exposed SHRs. Inflammatory markers of BALF, lung (pulmonary) and blood (systemic) were not affected. However, mRNA expression of hemeoxygenase-1, endothelin-1, endothelin receptors A and B, tissue factor, and plasminogen activator inhibitor showed a significant induction (~2.5-fold; p < 0.05) with endothelin 1 being the maximally induced factor (6-fold; p < 0.05) on the third recovery day in the lungs of UfCPs exposed SHRs; while all of these factors – except hemeoxygenase-1 – were not affected in cardiac tissues. Strikingly, the UfCPs-mediated altered BP is paralleled by the induction of renin-angiotensin system in plasma.

**Conclusion:**

Our finding shows that UfCPs exposure at levels which does not induce detectable pulmonary neutrophilic inflammation, triggers distinct effects in the lung and also at the systemic level in compromised SHRs. These effects are characterized by increased activity of plasma renin-angiotensin system and circulating white blood cells together with moderate increases in the BP, HR and decreases in heart rate variability. This systemic effect is associated with pulmonary, but not cardiac, mRNA induction of biomarkers reflective of oxidative stress; activation of vasoconstriction, stimulation of blood coagulation factors, and inhibition of fibrinolysis. Thus, UfCPs may cause cardiovascular and pulmonary impairment, in the absence of detectable pulmonary inflammation, in individuals suffering from preexisting cardiovascular diseases.

## Background

Epidemiological studies have identified exposure to elevated concentrations of ultrafine particles (UFPs; < 100 nm) in the air as a risk factor for the exacerbation of ischemic heart disease and congestive heart failure with specific physiological end points like arrhythmias, reduced heart-rate variability (HRV), elevated heart rate (HR) and atherosclerosis in adults [1-4]. However, the underlying pathophysiologcal mechanisms of airborne UFPs mediated cardiopulmonary mortality and morbidity are complex and remain to a large extent unexplored [5,6]. Although humans have been exposed to airborne UFPs throughout evolution, such exposure has increased dramatically over the last decades mainly due to increased emissions of combustion derived UFPs, e.g. from motor vehicles [7]. Thus, specific information about the molecular and pathophysiologcal mechanism involved in the cardiovascular impairments following exposure to UFPs is urgently required.

Dysfunction of the autonomic nervous system, i.e., an altered autonomic balance is amongst the recently discussed plausible biological mechanisms linking UFPs exposure with increased cardiovascular risk [2,8-10]. Exposure to airborne UFPs may cause a low-grade pulmonary inflammation by inducing the generation of reactive oxygen species and pro-inflammatory cytokines like TNF-α, IL-1, IL-6 [11]. This could be associated with increased plasma viscosity [12]; blood coagulability [13], vascular and endothelial dysfunction [14,15]. Furthermore, there are evidences that UFPs deposited in the lung gain access to the systemic circulation and translocate into extra-pulmonary organs, such as liver, heart, and brain [16,17]. This may disturb the blood coagulation balance by activating circulating platelets [18,19] but may also induce dysfunction in secondary target organs [20,21].

Previous studies from our laboratory [9] focussed on cardiovascular responses in young and healthy WKY (normotensive) rats following inhalation exposure to ultrafine carbon particles (UfCPs). The observed transient increase in HR associated with a decrease in HRV during exposure suggested an altered sympatho-vagal balance due to neural pathway activation in response to UfCPs inhalation [9]. Epidemiological studies have provided evidence that individuals with cardiovascular disease are at higher risk when exposed to elevated levels of ambient particles. Therefore, in this study we sought to investigate cardiopulmonary responses to UfCPs in spontaneously hypertensive rats (SHRs; 6 months), a well established animal model of human cardiovascular disease and assessed whether compromised animals (SHR) exhibit a different response pattern compared to what has been previously observed in healthy Wistar Kyoto (WKY) rats [9].

Compared to transient increases of HR during UfCPs exposure followed by a moderate pulmonary neutrophilic inflammation in WKY rats [9], SHRs demonstrated no sign of pulmonary neutrophilic inflammation or inflammatory mediator release, but obvious increases of HR and blood pressure (BP) for over a longer period of time after 24 h UfCPs inhalation (a lag of 1–3 days). Our mRNA expression and other biomarker data show that UfCPs exposure triggers distinct effects in the pulmonary tissue and also at the systemic level which can contribute to the observed cardiovascular impairments. Increases in BP along with induction of plasma renin-angiotensin system and increased expression for biomarkers of pulmonary oxidative stress; endothelial activation, and blood coagulation following exposure to UfCPs in cardiovascular compromised SHRs support the observed epidemiological findings of increased cardiovascular mortality as a result of exposure to peak ambient ultrafine particles concentrations.

## Results

### Cardiophysiological response *assessed by radio telemetry*

**Figure 1:**
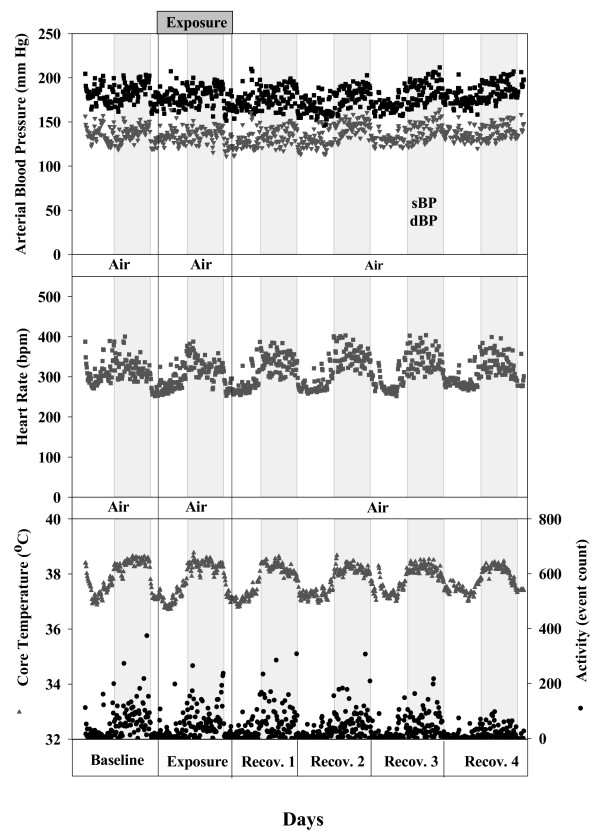
**Circadian rhythmicity of physiological and behavioural parameters of SHRs (n = 1)**. It shows the diastolic (dBP) and systolic (sBP) blood pressure, heart rate (HR), body temperature (T) and physical activity (Act) during basline, exposure and recovery periods. The dark period (night time) is indicated by the gray segment. Each data point of every parameter represents an average of 10-minutes data segments.

Figure 1 depicts 10-minutes data segments of systolic and diastolic arterial BP, HR, body temperature (T), and activity level (Act) of one rat for the time course of baseline (day 0), exposure (day 1), and recovery (day 2–5) periods. The data reflect typical circadian rhythmicity of physiological and behavioural (activity) parameters of SHRs as characterized by elevated BP, HR, T, and Act values during the dark periods.

**Figure 2:**
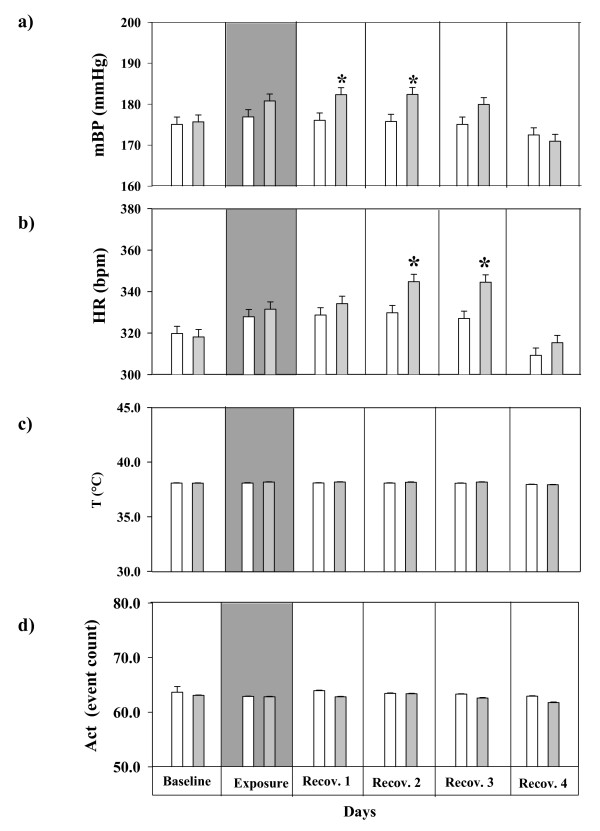
**a Changes in mean blood pressure (mBP) associated with filtered air (control)/ultrafine carbon particles (UfCPs, exposed) exposed SHRs.** mBP increased by 6 mmHg, (4%) on the first and second day of recovery in the UfCPs exposed groups compare to control SHRs. * indicates significant difference of mBP (p < 0.05) between exposed (gray)  and control (white)  SHRs. 2b: Changes in heart rate (HR) of SHRs associated with filtered air (control)/ultrafine carbon particles (UfCPs; exposed) exposure. HR responded with a lag of one day, it increased on the second and third day of recovery by 17 bpm (5%) in UfCPs exposed SHRs, and reached baseline values on the fourth day of recovery. * indicates significant difference of HR (p < 0.05) between exposed (gray) and control (white) SHRs. 2c: Changes in body temperature (T) of SHRs associated with filtered air (control)/ultrafine carbon particles (UfCPs; exposed) exposure. 2d: Changes in activity (Act) of SHRs associated with filtered air (control)/ultrafine carbon particles (UfCPs; exposed) exposure.   The vertical bars exhibit arithmetic mean values (mean ± SE) of control (white; n = 7) and exposed (gary; n = 7) groups. Each bar represents a combined mean value of: (72 10-minutes segments/12h dark periods/rat) × 7 rats.

Comparison of baseline values between 6 and 7 months old SHRs indicate that cardiac performance was not altered by the 4 week time gap between control (6. months) and exposure (7. months) conditions (Figures 2 a–d). Baseline values of mBP (control/exposure: 176 ± 1.2/177 ± 1.2 mmHg), HR (control/exposure: 320 ± 3.5/318 ± 3.4 bpm), T (control/exposure: 38.1 ± 0.03/38 ± 0.03°C) and Act (control/exposure: 63.6 ± 1.4/63 ± 0.06 event count) remain unchanged by the 4 week time gap between control and exposure conditions.

Figures 2a and 2b display mean values (± SE) for mean arterial blood pressure (mBP) and HR during dark periods of baseline, exposure and recovery. Following 24 h exposure to UfCPs mBP increased by about 6 mmHg, (control/exposure: 176 ± 2.0/182 ± 2.1 mmHg; about 4%, p < 0.05) on the first and second day of recovery in exposed SHRs, and returned to baseline levels on the fourth day of recovery. This is due to the concurrent increase in systolic (control/exposure: 194 ± 2.8/204 ± 2.5 mmHg) and diastolic BP (control/exposure: 158 ± 1.5/160 ± 1.8 mmHg) with the systolic being more pronounced. In comparison to the BP response, HR responded with a lag of one day, it increased on the second and third day of recovery by about 17 bpm, (control/exposure: 328 ± 3.5/345 ± 3.5 bpm; about 5%, p < 0.05) and reached baseline values on the fourth day of recovery. UfCP exposure did not affect body temperature or activity levels of the animals. Both remained unaffected in UfCPs exposed SHRs compared to their control (Figures 2c and 2d).

**Figure 3:**
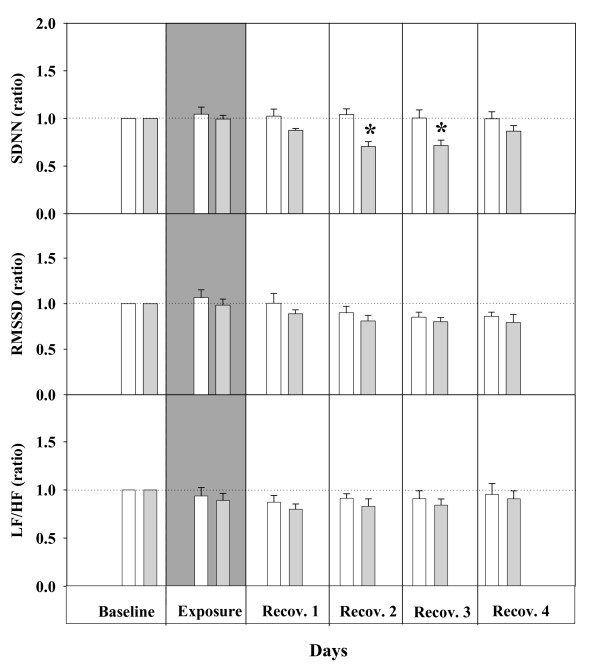
**Relative Changes in time domain and frequency domain measures of heart rate variability (HRV) of SHRs associated with filtered air (control)/ultrafine carbon particles (UfCPs; exposed) exposure**. Bars represent arithmetic mean values ± SE of control (white bars; n = 7) and exposure groups (gray bars; n = 7): (12 5-minutes segments/12h dark period/rat) × 7 rats. Dotted horizontal lines are used to highlight the relative changes. HRV, paralleled changes in HR was decreased by about 30% in UfCPs exposed SHRs during the recovery periods (second and third day). *: indicates significant differences of HRV (p < 0.05) between exposed (gray) and control (white) SHRs. SDNN: standard deviation of normal to normal (NN) intervals. RMSSD: square root of the mean of squared differences between adjacent NN intervals. LF/HF: ratio of the absolute powers in the low-frequency (LF: 0.20 Hz to 0.75 Hz) and high-frequency bands HF: 0.75 Hz to 2.5 Hz).

The standard deviation of all normal adjacent sinus intervals (SDNN), a measure of the overall heart-rate variability (HRV), was decreased by about 30% (p < 0.05) during the recovery period on the second and third day (Figure 3). The square root of the mean of squared differences between adjacent normal to normal intervals (RMSSD) and the low-frequency to high-frequency ratio (LF/HF), showed a comparable response as SDNN, but failed to be statistically significant (Figure 3). No individual changes in absolute LF (baseline: 21.5 ± 1.13 nu; second recovery day: 17.6 ± 1.3 nu; third recovery day: 18.1 ± 0.89 nu) and HF (baseline: 70.2 ± 1.9 nu; second recovery day: 67.8 ± 2.8 nu; third recovery day: 72.5 ± 1.6 nu) power have been observed. The observed transient increase in HR associated with overall decrease in HRV (SDNN) suggests an altered sympatho-vagal balance in response to UfCPs inhalation.

### Pulmonary inflammatory response

#### BALF and lung

BALF derived parameters obtained on first and third recovery day showed no signs of UfCPs exposure related inflammatory response in the lungs. Cell numbers and cell differentials of the BALF (Total cell: control/exposed: 3.7 ± 0.2/4.7 ± 0.2; PMN: control/exposed: 0.3 ± 0.1/0.4 ± 0.1) were not affected in exposed SHRs. BALF protein (control/exposed: 137 ± 32/135 ± 34 μg/ml), albumin concentrations (control/exposed: 18 ± 5/15 ± 5 μg/ml), and γ-Glutamyltransferase (GGT; control/exposed: 4.9 ± 0.4/4.8 ± 0.5 U/l) activity were used as markers of pulmonary capillary leakage or pulmonary cell membrane integrity; whereas N-acetyl glucosaminidase (NAG; control/exposed: 5.3 ± 0.5/5.8 ± 0.7 U/l) activity in BALF indicate macrophage phagocytic ability. None of these markers from BALF showed any significant changes in exposed SHRs. The pulmonary cytokine IL-6 in BALF samples of exposed SHRs showed a slight, 10% increase (control/exposed: 83 ± 6.2/92 ± 4.1 pg/ml), but differences were not statistically significant. Furthermore, transcript profiling markers associated with inflammation (MIP-2, TNF-α), were assessed from the lung tissues on first and third recovery day. Corresponding to the findings in BALF, expression of MIP-2 and TNF-α were not significantly altered on both days (data not shown).

#### Pulmonary histopathology

Pulmonary histopathology analysis also revealed no signs of pulmonary inflammation in the UfCPs exposed animal group (data not shown).

### UfCPs-mediated direct effect on pulmonary and cardiac tissue

#### Pulmonary mRNA expression

**Figure 4:**
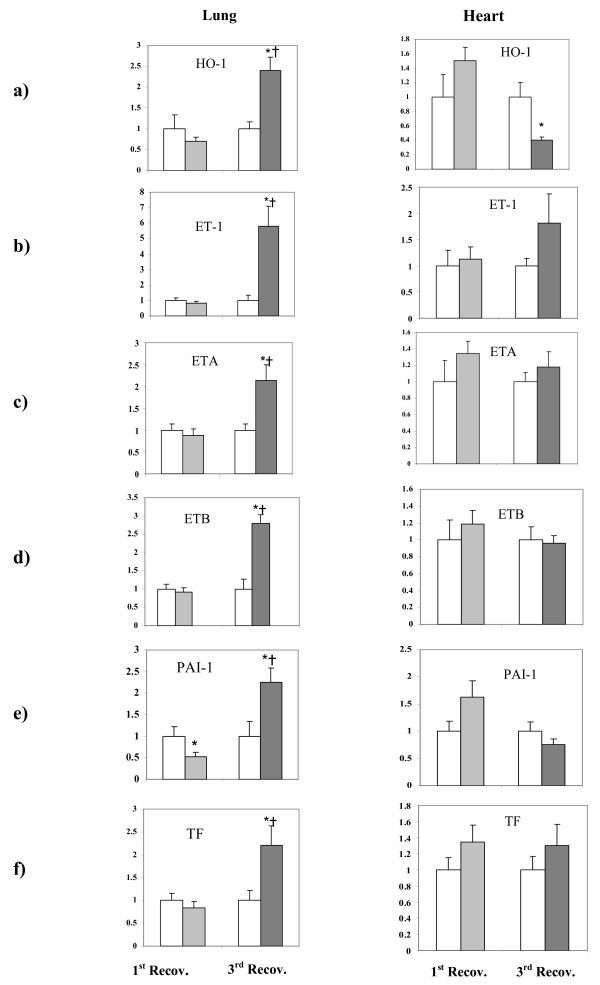
**Changes in the lung and heart transcript levels of haemoygenase-1 (HO-1; a), endothelin-1 (ET-1; b), endothelin receptor A (ETA; c), endothelin receptor B (ETB; d), plasminogen activator inhibitor-1 (PAI-1; e) and tissue factor (TF; f) in control (filtered air) and ultrafine carbon particles (UfCPs) exposed SHRs on the first and third recovery day**. Real time qRT-PCR was performed and the comparative C_T _method was used for the quantification of fold change. Bars represent arithmetic mean values ± SE of control (white bars; n = 6) and UfCPs exposed SHRs on first (light gray bars; n = 6) and third recovery day (dark gray bars; n = 6). Lung expression of all other markers (except PAI-1) was not affected on the first day of recovery (light gray) – but showed significant inductions on the third day of recovery (dark gray) by factors of about 2.5, except for ET-1 which increased about 6-fold. *: Significant (p < 0.05) difference in expression level between exposed animals compare to the corresponding control: †: Significant (p < 0.05) difference in expression level between exposed SHRs on first (light gray) and third (dark gray) recovery day.

Transcript profiling markers associated with oxidative stress (hemeoxygenase-1: HO-1), endothelial activation (endothelin-1: ET-1; endothelin recptor A and B: ETA and ETB), and coagulation factors (tissue factor: TF; plasminogen activator inhibitor-1: PAI-1) were assessed from lung tissues of control and UfCPs exposed SHRs on first and third recovery day (Figures 4 a–f, left column) using quantitative real time Polymerase Chain Reaction (qRT-PCR). All of these markers showed a significant induction (~2.5-fold; p < 0.05) on the third recovery day with ET-1 being the maximum induced factor (6-fold; p < 0.05). Although PAI-1 expression was slightly decreased on first day of recovery, but at third recovery day its expression was increased over 2-fold in the lung.

#### Cardiac mRNA expression

To assess whether pulmonary and cardiac tissue exhibited a comparable response, HO-1, ET-1, ETA, ETB, PAI-1 and TF were also measured in the cardiac tissues on the first and third day of recovery. Compared to the lung only slight but non significant exposure related increases were detected in the cardiac tissues. Strikingly, HO-1 was repressed by over 2-fold in the heart in contrast to its more than 2-fold induction in the lung on the third day of recovery (Figures 4 a–f, right column).

#### Cardiac histopathology

Histological analysis revealed fibrotic foci (typical for SHRs), but no signs of inflammation or cardiomyopathy following exposure to UfCPs (data not shown).

### Systemic response

#### Acute phase proteins

To assess whether UfCPs exposure induces an inflammatory response at the systemic level the acute phase reactants, C-reactive protein (CRP; control/exposed: 92 ± 3.8/94 ± 3 μg/ml)), haptoglobin (HP; control/exposed: 300 ± 17/309 ± 20 mg/dl)) and fibrinogen (control/exposed: 190 ± 11.4/198 ± 5.2 mg/dl) were determined from serum and plasma. None of these markers revealed any significant changes in exposed SHRs compared to the control groups.

#### Haematology

The complete blood cell count [total red and white blood cell, haematocrit, platelets, polymorphonuclear neutrophil (PMN), and lymphocytes] was assessed on first and third day of recovery. Most of the blood parameters were not affected by UfCPs exposure (Table 1), but cell differentials revealed significant increase (p < 0.05) in the fraction of neutrophil (control/exposure: 30 ± 2.8/43.3 ± 2.2%) and lymphocyte (control/exposure: 43.3 ± 3.1/56.3 ± 2.9%) on the first day of recovery (Table 1).

**Table 1:** Haematological analysis of filtred air (control;n = 8) and ultrafine carbon particles (UfCPs;n = 8) exposed SHRs on first and third recovery day.

**Parameters**	**First recovery day**		**Third recovery day**	
	
	**Control**	**Exposed**	**Control**	**Exposed**
RBC (×10^3 ^cells/μl)	7.92 ± 0.36	9.6 ± 0.3	8.29 ± 0.22	9.41 ± 0.11

WBC (×10^3 ^cells/μl)	6.19 ± 0.4	5.79 ± 0.5	8.7 ± 0.9	6.6 ± 0.7

PLT (×10^3 ^cells/μl)	436 ± 30.2	443.8 ± 48.4	611 ± 22	582 ± 24

Haematocrit (%)	44.8 ± 0.8	43.8 ± 2.1	46 ± 1.2	44 ± 0.4

Thrombocrit (%)	0.51 ± 0.04	0.39 ± 0.05	0.6 ± 0.02	0.45 ± 0.04

Neutrophil (%)	30 ± 2.8	*43.3 ± 2.2	44 ± 1.5	48 ± 2.04

Lymphocyte (%)	43.3 ± 3.1	*56.3 ± 2.9	45 ± 1.5	50 ± 1.9

* Significant difference (p < 0.05) between control and exposed animals RBC: Red blood cells;

WBC: white blood cells; PLT: platelet.

#### Renin/Angiotensin

**Figure 5:**
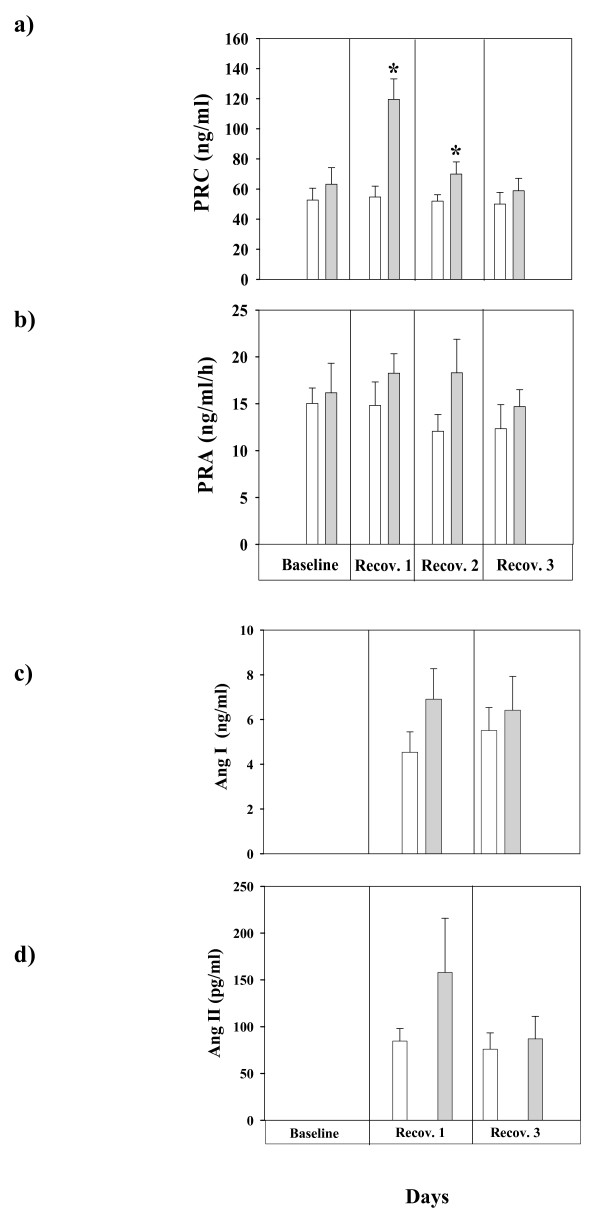
**a: Plasma renin concentration (PRC) of control (filtered air) and ultrafine carbon particles (UfCPs) exposed SHRs at several time points: before exposure (baseline), first, second and third recovery days.** Bars represent arithmetic mean values &#177 SE of control (white bars; n = 8) and exposure groups (gray bars; n = 8). Significant increases of PRC were detected on the first and second day of recovery. *: Significant difference (p < 0.05) of PRC between exposed and control SHRs (n = 8/8; control/exposed) on first and second recovery day. 5b: Plasma renin activity (PRA) of control (filtered air) and ultrafine carbon particles (UfCPs) exposed SHRs at several time points: before exposure (baseline) and first, second and third recovery days. Bars represent arithmetic mean values &#177 SE of control (white bars; n = 8) and exposure groups (gray bars; n = 8). 5c: Plasma angiotensin I (Ang I) concentration of control (filtered air) and ultrafine carbon particles (UfCPs) exposed SHRs on first and third recovery day. Bars represent arithmetic mean values &#177 SE of control (white bars; n = 8) and exposure groups (gray bars n = 8). 5d: Plasma angiotensin II (Ang II) concentration of control (filtered air) and ultrafine carbon particles (UfCPs) exposed SHRs on first and third recovery day. Bars represent arithmetic mean values &#177 SE of control (white bars; n = 8) and exposure groups (gray bars n = 8). Increased values of angiotensin II were detected on the first day of recovery but differences are not statistically significant.

To assess whether the renin-angiotensin system (RAS) is involved in the observed cardiovascular response (BP and HR, Figures 2a &2b) following UfCPs exposure we analyzed plasma renin concentration and activity (Figures 5a &5b) at several time points from blood samples of the caudal vein (blood B; Table 2). Measurements of renin concentration and activity as well as angiotensin (I and II) concentrations were assayed employing specific radioimmunoassay [22]. Significant (p < 0.05) increases of plasma renin concentration were detected on the first and second day of recovery. Levels returned to baseline values at the third day of recovery (Figure 5a). However the activity of renin was not affected by UfCPs exposure (Figure 5b). Additionally, Ang I and II concentrations were also determined on the first and third day of recovery in blood samples from the abdominal aorta (blood A; Table 2). A tendency of increased Ang I and II concentrations were detected on the first day of recovery in exposed animals; however the differences remained statistically unaltered (Figure 5c & d).

**Table 2:** Experimental design of cardiophysiological, pulmonary and systemic response

**Study**	**Day -2**	**Day -1**	**Day 0**	**Day 1**	**Day 2**	**Day 3**	**Day4**	**Day5**
	**Adaptation**		**Baseline**	**Exposure**	**Recovery**			

Cardiophysiological Response*								

Telemetry (n = 7)	**Acclimatization**		**√**	**√**	**√**	**√**	**√**	**√**
	
	**No change**				**BP↑**	**BP↑ HR↑**	**HR↑**	

**Pulmonary response**								

BALF (n = 6)					**√**		**√**	

Lung tissue (n = 6)					**√**		**√**	

Pulmonary histopathology (n = 2)					**√**		**√**	

**Systemic Response**								

Blood A (n = 8)					**√**		**√**	

Blood B (n = 8)					**√**	**√**	**√**	

Heart tissue (n = 6)					**√**		**√**	

Cardiac histopathology (n = 2)					**√**		**√**	

* Cardiophysiological response by telemetry: individuals served as their own control. Pulmonary and systemic response: number of exposed animals is given. A similar number of animals was exposed to filtered air and served as controls. Total animal number: 39. **Blood A**: blood collected from retro arbitral sinus and abdominal aorta, **Blood B**: blood collected from caudal vein. **BP↑, HR↑**: blood ressure or heart rate significantly elevated after UfCPs exposure, respectively. **√**: performed.

## Discussion

Exposure to ambient air pollution has been associated with increased cardiovascular morbidity and mortality [1-4]. The currently emerging occupational and environmental burden of UFPs from the rapidly developing field of nanotechnology evokes concerns about the health effect of engineered nanoparticles (ultrafine) worldwide. Our exposure study specifically addresses the action of laboratory generated, airborne-like UFPs in the respiratory and the cardiovascular system as very little is known about the potential adverse effects and the underlying pathomechanisms. Findings from this work indicate increases in BP associated with striking activation of plasma rennin angiotensin system together with increases in circulating white blood cells and increased expressions for markers of pulmonary endothelial activation, blood coagulation, and oxidative stress in cardiovascular compromised UfCPs-exposed rats. Because these changes occur at UfCPs concentration which does not produce apparent pulmonary inflammation our results support epidemiological associations of elevated particle levels and cardiovascular impairment in individuals with preexisting diseases.

Compared to transient increases of HR and pulmonary neutrophilic inflammation during UfCPs exposure in WKY rats [9]; inhalation of UfCPs results in a prolonged increase in HR and BP in SHRs with a lag of 1–3 days, but no pulmonary neutrophilic inflammation. Lack of neutrophilic influx in association with no significant increase in MIP-2 and/TNF-α mRNA expression in SHRs suggests that the observed cardiovascular impairment is relatively independent of an apparent inflammatory response although an association to other observed pulmonary and systemic effects can not be ruled out. The lack of neutrophilic inflammation is very unusual as we have noted this happen in WKY rats similarly exposed to UfCPs [9]. However; it is not totally surprising in SHRs as it has been shown in some studies that these rats (SHRs) require a greater insult to initiate inflammation than WKY [23]. Although there is no neutrophilic inflammation in the lung but significant induction of HO-1, PAI-1, TF and other markers such as endothelin-1 is an indicator of pulmonary oxidative stress and injury. Considering this observation we hypothesize that SHRs (hypertensive individuals) are at higher risk and more susceptible or vulnerable to cardiovascular impairments following exposure to UFCPs compared to healthy WKY as no pulmonary injury or coagulative changes were observed in WKY similarly exposed to UfCPs [9].

A series of studies have shown that following exposure UFPs gain rapid access to pulmonary epithelial, interstitial and endothelial cells and can also be translocated from peripheral lungs to systemic circulation and extra-pulmonary organs in a considerable amount [17,24,25]. Therefore, it is probable that the detected cardiovascular impairments may primarily be due to the direct interaction of UfCPs with pulmonary tissue and at systemic level results in oxidative stress, activation of endothelial system, induction of blood coagulation factors, and inhibition of fibrinolysis together with induction of renin-angiotensin system at a systemic level.

### UfCPs burden in the lungs and at systemic level

Responses to UfCPs were studied considering particle number and mass concentrations, which approximate peak ambient particulate matter burdens [26,27]. Assuming a mean minute ventilation of 214 ml minutes^-1 ^[28] the rats in this study inhaled 308 liter of aerosol (172 μg m^-3^) in 24 h, resulting in an inhaled cumulative dose of approximately 53 μg UfCPs. It has been shown [29] that inhalation exposure of rat to ultrafine gold particle (mass median diameter: 49 nm) under similar conditions results in an alveolar deposition rate of 20%. Considering this deposition rate, the alveolar burden of UfCPs in our present experiment is ~10.6 μg or 5.5 × 10^11 ^particles. With respect to our previous studies conducted in rats [17,24] about 20% of the deposited dose, i.e. 2 μg/rat, is supposed to be rapidly translocated into pulmonary tissues and may exert direct intracellular effects in these tissues. According to the translocation studies [17] much less than 1 μg may be systemically available and less than 0.1 μg may reach the heart. These dose estimates corresponds to the dose that a healthy human will accumulate over one year when being exposed to common ambient levels of insoluble UFPs [30], i.e. 3 × 10^11 ^UFPs will accumulate in the lungs and 6 × 10^8 ^UFPs in each of the secondary target organs (the estimate is based on a particle concentration of 1 × 10^3 ^cm^-3^, a daily inhaled gas volume of 1 × 10^4 ^l/d, and a deposition fraction in the peripheral lung of 0.3). However, ambient UFP concentration near busy roads may exceed the assumed particle number concentration substantially up to a factor of 100 [31] and the deposition fraction may be higher in individuals suffering from respiratory diseases, e.g. ~70% in asthmatics [32]. This suggest that the biological responses detected in the present study may be related to peak ambient ultrafine particle exposures to which humans may incidentally or accidentally be exposed to.

### Cardiophysiological performance

The cardiophysiological response in SHRs was characterized by a prolonged increase in BP and HR by about 5% during first to third day of the recovery period (p < 0.05). The extent of HR increase in UfCPs-exposed SHRs is consistent with other studies [33,34] and that of our previous study [9] showing a significant elevation of HR by ~5% in WKY rats exposed to a comparable UfCPs concentration (180 μg m^-3^). However, in our previous study the normotensive (WKY) rats showed only increased HRs during the exposure period and did not exhibit any changes in BP. Similarly Gordent et al. [35] reported small but significant increases of HR (approximately 5%) in healthy rats during 6 h nose-only exposures to concentrated ambient particles (132 μg m^-3^-184 μg m^-3^). In SHRs about 8% increase of HR has been observed at much higher diesel particle concentrations [1000 μg m^-3^; [33]. Furthermore, the mild but significant BP increase is comparable with the physiological endpoints from F-344 rats after exposure to urban ambient particles [36]. Overall, BP and HR changes are admittedly small; however, with respect to the UfCPs exposure level selected, they are reasonable and likely to occur also in humans. Comparison of response patterns in healthy and compromised animals suggests that hypertensive animals are more susceptible to UfCPs particle exposure than normotensive. We detected a significant increase in BP and HR in SHRs over a period of 3 days after exposure to UfCPs. Whereas in other studies with uncompromised animal model, alteration of cardiovascular perfomance (HR and/or BP) was noted during the exposure and the values reach baseline levels rapidly after exposure [9,33,35].

### Pulmonary and systemic inflammatory responses

A series of epidemiological studies have shown that exposure to ambient ultrafine particles is associated with pulmonary inflammation caused by deposition of particles in the alveoli [10,37]. Particle exposure may results in local and systemic inflammatory responses [6,11] and ultimately may lead to an activation of the coagulatory system [18,19], increased plasma viscosity [12], vascular and endothelial dysfunction [14,15]. Therefore, we assessed pulmonary and systemic inflammatory responses in order to shed light on pathophysiological pathways, which might be the plausible cause of UfCPs induced cardiovascular effects. No apparent inflammatory response has been detected in pulmonary tissue but a low grade inflammatory reaction cannot be ruled out. Plasma level of acute phase proteins such as HP and CRP were not affected by UfCPs exposure, but the observed small increases in the fraction of neutrophils and lymphocytes following 24 h exposure to UfCPs may indicate a small degree of systemic inflammation. Similar observations have been made by others and such phenomenon are suggested to contribute to the progression of atherosclerosis, hypertension and increased the risk of cardiopulmonary disease [38,39]. The precise mechanism(s) of how systemic impairment is produced by inhalation of UfCPs and its consequence on cardiophysiology need to be explored.

### Pulmonary and extra-pulmonary effects unrelated to inflammation

The pulmonary response of exposed SHRs is characterized by a significant induction of HO-1 (~2.5-fold), a sensitive marker for UfCPs mediated oxidative stress [40,41], on the third day of recovery. The induced HO-1 is an indicator of a host defense mechanism for oxidative stress and is likely to be directly activated by UfCPs which have been shown to provide a substantial oxidative potency [42]. The observed induction of PAI-1 (~2.5-fold) can be considered to be a downstream effect of particle induced oxidative stress [43]. Increased levels of PAI-1, have been recognized as hallmarks of impaired endothelial function and are a common denominator of increased risk for cardiovascular disease [44]. PAI-1 is known to be an inhibitor of fibrinolysis and regulator of vasoactivity [45], thus, increased levels of PAI-1 mRNA expression may also be involved in the observed increase in BP. Further, an increased TF expression (~2.5-fold) was detected in the lungs. Increased TF levels have been related to an increased risk of cardiac events because induction of TF is highly correlated with thrombogenesis and endothelial dysfunction [46,47]. Activation of TF, the extrinsic coagulation pathway, in association with impaired fibrinolysis via PAI-1 activation suggests that UfCPs exposure induces endothelial dysfunction and activates the coagulatory pathway, both of which are correlated with overall cardiovascular risk [45-47].

Additionally, induction of ETA and ETB (~2.5-fold) along with a 6-fold induction of pulomonary ET-1 further supports the notion of an UfCPs induced endothelial dysfunction in the pulmonary circulation. The simultaneous increase in ETA and ET-1 indicates a synergistic effect because ET-1 contributes to endothelial dysfunction predominantly via ETA receptor stimulation [48]. Potential health implications are obvious since several reports imply that both ETA and ETB are contributing to ET-1 induced hypertension [49,50]. Moreover, up-regulation of ETB receptors is most notable in heart failure, hypertension and in artherosclesis formation [51].

Hence, the observed induction of the endothelin system (ET-1, ETA and ETB) and increased expression of coagulatory factors (PAI-1 and TF) in pulmonary tissue suggests that UfCPs exposure triggers important pathophysiological pathways in the lungs which have been associated with impaired cardiovascular performance and an increases risk for cardiovascular events.

Interestingly, in the heart of UfCPs exposed SHRs, the only marker being affected was the oxidative stress-inducible defense enzyme HO-1. Expression of HO-1 mRNA was more than 2-fold repressed in contrast to its up-regulation in the lung tissue. Our data do not allow to provide a final explanation for this observation, but under hypoxic conditions HO-1 mRNA is reported to be induced by a number of studies while others found a repression [52]. It was suggested that the differential regulation and the different expression levels of HO-1 represent an adaptation or unrecognized defense strategy to stress and that the response can differ depending on cell and tissue type [52,53]. Accordingly, we may value the different regulations observed for HO-1 in lung and cardiac tissue in the present study. However, we have to specify that we do not have any hint that UfCP exposure results in hypoxic conditions in cardiac tissue.

### Systemic effects associated with cardiopulmonary impairments

The most interesting UfCPs-mediated systemic effect is the close association of increased BP in exposed SHRs (Figure 2a) with a significant increase of circulating PRC at the same time points (Figure 5a). The tendency of increased Ang I and II concentrations on the first day of recovery further supports the notion that the RAS is primarily involved in the UfCPs-mediated BP increase detected in exposed SHRs [54-56]. On the other hand, the enzymatic activity of renin appears not to be affected by UfCPs exposure. Renin is a rate-limiting enzyme determining the overall RAS activity. It converts plasma angiotensinogen to Ang I, which is subsequently converted to Ang II, one of the most powerful vasoconstrictor. The RAS system is typically associated with the perfusion of the kidney, however, several recent studies reported that local RAS exist which is physiologically active in different tissues like lung, heart, kidney, or brain [55,57,58]. Besides regulating regional perfusion, local RAS may also play an important role in regulating systemic blood pressure [55,57,58]. Therefore, induced RAS in plasma might be due to direct effect of UfCPs in the pulmonary tissue, resulting in local RAS activation and systemic blood pressure elevation.

## Conclusion

We have shown that inhalation of UfCPs at concentrations reflecting peak ambient exposures results in a moderate (about 5%) increase in HR and BP in SHRs with a lag of 1–3 days which is paralleled by the induction of renin-angiotensin system in plasma, potentially being due to an activation of the local pulmonary rennin-angiotensin system. Specifically in pulmonary tissue markers of oxidative stress (HO-1), the endothelin system (ETA, ETB and ET-1) and the coagulation system (PAI-1 and TF) were found to be activated by UfCPs exposure. Since various inflammatory markers in pulmonary tissues (TNF-α, MIP-2; IL-6) were not affected and neutrophil cell recruitment was not observed, the UfCPs induced effects appear to be unrelated to a traceable pulmonary inflammation. At the systemic level different inflammatory markers (acute phase protein: CRP, HP) were also not affected, but the observed increases in neutrophils and lymphocytes following 24 h exposure to UfCPs suggest a possible low degree of systemic inflammation. Therefore, our findings imply that UfCPs exposure at levels below detectable pulmonary inflammation triggers distinct effects in pulmonary tissues and at systemic levels that can promote further cardiovascular impairment in SHRs. Since dose estimates revealed that the burden of UfCP delivered to the rats exceeds that caused by common urban levels in humans the observed biological effects may only be related to peak ambient ultrafine particle exposures, e.g. at busy rods, to which humans are incidentally or accidentally be exposed to. Based on the response detected in SHRs – as a model for impaired cardiovascular individuals – as well as those observed in healthy WKY rats [9] we reciprocate the epidemiological findings that predisposed individuals are at higher risk and more susceptible to cardiovascular impairments following exposure to UfCPs than healthy ones.

## Methods

### Animals

Male spontaneously hypertensive rats (SHR; 6 month) were used for the present study. Animals were housed under filtered air and specific pathogen free (SPF) conditions at a mean temperature of 22 ± 2°C, a mean relative humidity of 50 ± 5%, and a 12 h light-dark cycle (6 a.m. to 6 p.m. light on) with pelleted feed and filtered water being supplied *ad libitum*. Experimental protocols were approved by the Animal Care and Use Committee of the HelmholtzZentrum München – German Research Center for Environmental Health and by the Bavarian Animal Research Authority (211-2531-88/2001).

### Ultrafine carbon particle generation and whole body exposure chamber

The methodology of UfCPs generation and the setup of the whole body exposure system for rodent have been previously described [9,59]. UfCPs showed a monomodal number distribution in the exposure chamber with a median particle size ± arithmetic SD of 31 ± 0.3 nm. Measured mass and number concentration was 172 μg m^-3 ^and 9× 10^6^cm^-3^, respectively. This translates into a surface area concentration of 0.139 m^2^(particle) m^-3 ^(air) because the mass specific surface area (according to the BET method) of the UfCPs was determined to be 807 m^2 ^g^-1^. Based on the polydispersity of the particle distribution (geometric standard deviation is 1.51) a median mass diameter of 46 nm is calculated.

### Experimental design

Table 2 provides an overview of the experimental design. Primarily, the cardiophysiological responses, i.e. effects on HR and BP were measured in 7 SHRs following 24 h UfCPs inhalation exposure using a radio telemetry system. Since BP and HR were increased on first to third day of recovery, the subsequent exposures were conducted in additional, non-telemetry SHRs to obtain blood, BALF and tissue samples from the first and third day of recovery. Each exposure used 16 SHRs, 8 animals were exposed to filtered air (controls) while the other 8 animals were exposed to UfCPs for 24 h (exposed). In the first study, animals were sacrificed in the morning of the first day of recovery. Prior to BALF collection, blood samples (blood A) of 8 SHRs were collected from retro orbital sinus for analysis of haematological parameters and from the abdominal aorta for analysis of biomarkers. Six animals were used to collect BALF and tissue samples (heart and lung) for further assessment of pulmonary and systemic response, the remaining 2 SHRs of each group were used for pulmonary and cardiac histopathology. Animal distribution and sample collection of the control group was similar to that of the exposed group.

In the second inhalation study, SHRs were sacrificed in the morning on third day of recovery. Blood sampling from retro orbital sinus and abdominal aorta (blood A), BALF and tissue samples collection, as well as cardiac histopathology were carried out as described above. In addition, 400 μl of blood from the caudal vein of each animal (blood B) was collected in the morning before exposure (base line) and on the first, second and third day of recovery to assess plasma renin concentration and activity.

### Cardiophysiological analysis by radiotelemetry

#### Exposure protocol

Cardiophysiological response prior to and following inhalation exposure to UfCPs was performed on 7, 6 months old SHRs (360 ± 11 g) by using radio telemetric system as described in our previous study [9] (Dataquest A.R.T; Data Sciences International D.S.I., St. Paul MN, U.S.A). The implantation of telemetric devices into the peritoneal cavity of animals was performed as previously described [9]. All animals exhibited rapid post surgical recovery, with resumption of normal food and water intake within 24 h of surgery. They returned to presurgical body weight (excluding the weight of the implant) on average within 3–4 days and did not exhibit any signs of post surgical complications. After 10 days of post surgical recovery, the animals were acclimatized in the exposure chamber for two days (day -2 and day -1). We have observed that following 2 days of acclimatization in the exposure chamber prior to the actual data recording cardiovascular response (BP and HR) reaches to its baseline values. Data recording was then initiated and continued for six days (Figure 1), that included a baseline reading (day 0), exposure (day 1), and recovery period readings (days 2–5).

In this study the individual animals served as their own controls. Applying the above defined exposure protocol animals were primarily exposed to filtered air (control) and 4 weeks later to UfCPs by whole-body exposure. A time gap of 4 weeks was chosen to ensure elimination of any possible effects of clean air exposure. Comparison of baseline values between 6 and 7 months old SHRs indicate that cardiac performance was not altered by the 4 week time gap between control and exposure conditions (see baseline values in results). Moreover, no significant changes of cardiovascular performance (mBP and HR; Figures 2a and 2b) was noted in SHRs following exposure to filtered air.

#### Animal preparation, data acquisition and analysis using radio telemetry system

The implantation of telemetric devices into the peritoneal cavity of animals (n = 7), the radio telemetric data acquisition and analysis were performed as described previously [9].

Briefly, arterial BP, HR, body core temperature (T), and physical activity (*Act*) of SHRs were continuously collected over 24 h/day, throughout baseline, exposure and recovery periods. Systolic (sBP), diastolic (dBP), and mean (mBP) arterial blood pressure were determined from the BP tracings on a beat to beat basis. The data of each animal were then processed to obtain 10-minutes average segments per rat for each of the measured parameters (Figure 1). For the final data analysis, we only have considered the values of each parameter from the 12 h dark period (6 p.m. to 6 a.m.), as animals are more active during the night time. Thereby, 72 consecutive values of 10-minutes data segments were obtained per rat per day and per parameter. Since we did not observe a time dependency of particle associated effects during the 12 h period in each of the exposed animals, mean values were used for further data processing. For all of the measured parameters, we averaged the 72 values obtained for each day resulting in one mean value per parameter per rat and per day. Based on these values, group mean averages were calculated on a daily bases for the whole study and were used for statistical comparison between filtered air (control) and UfCPs exposed SHRs.

For heart-rate variability (HRV) analysis, a different procedure has to be applied [9]. For the 12 h dark periods, one 5-minutes ECG segment per hour was randomly selected and used for further HRV analysis. For each of these 5-minutes segments the standard deviation of all adjacent normal sinus NN intervals (SDNN) was determined as a measure of the overall HRV. In addition, the square root of the mean of squared differences between adjacent normal to normal intervals (RMSSD) and the low-frequency to high-frequency ratio (LF/HF), reflecting the balance of cardiac parasympathetic tone and sympathetic activity, respectively, were determined. Further data processing to obtain daily averages for each of the rats and group averages followed the procedure described above for the other parameters.

### Assessment of UfCPs-mediated pulmonary inflammatory response

#### BALF and lung

BALF analysis was performed as described in our previous study [9]. In brief, one aliquot of whole BALF (n = 6) was used for determining total cell counts (Coulter Counter; Coulter, Inc., Miami, FL), and a second aliquot was centrifuged (Cytospin 2; Shandon, Astmoor, UK) to counts cell differential. Macrophages, polymorphonuclear cells (PMNs, or neutrophil), eosinophil, and lymphocyte were counted using light microscopy (over 200 cells counted per slide). The remaining BALF was centrifuged (1500 × g) to remove cells, and the supernatant fluids were analyzed for protein, albumin concentration and γ-Glutamyltransferase (GGT), N-acetyl glucosaminidase (NAG) activity as potential biological markers for pulmonary capillary leakage and lung injury [60]. Furthermore, transcript profiling markers associated with pulmonary inflammation (MIP-2, TNF-α), were assessed from the lung tissues (n = 6) using real-time RT-PCR (see gene expression analysis).

#### Pulmonary histopathology

The left lung of each non lavaged animal (n = 2) was infused via left main bronchus by 4% buffered formalin at 20 cm water pressure for 20–30 minutes. The main bronchus was then tied and the lung was submerged in fixative until processing for histology. Paraffin blocks were prepared from dehydrated tissues and 3- to 4-μm sections were stained with hematoxylin and eosin for light microscopic evaluation of the pulmonary tissues [9].

### Assessment of UfCPs-mediated effects on pulmonary and cardiac tissue

#### Gene expression analysis

For gene expression analysis lung and heart tissues were collected from each animals (n = 6) immediately after BALF collection, placed in vials and flash frozen in liquid nitrogen. They were then stored at -80°C until extraction of RNA.

#### RNA isolation

Total RNA was extracted from these tissue samples using the RNeasy (lung) or RNeasy fibrous tissue (heart) kits and protocols obtained from Qiagen. RNasin Plus (Promega), a broad spectrum RNase inhibitor, was added to each sample immediately after isolation from the tissue. Concentration and purity of the RNA samples were determined with the NanoDrop ND-100 spectrophotometer (NanoDrop Technologies). Aliquots of each sample were diluted to a concentration suitable for PCR and the stock samples and dilutions were stored at -80°C.

#### Gene expression assays

Relative quantification of gene expression was determined using real-time qRT-PCR on the Applied Biosystems Inc. model ABI 7900 HT Sequence Detection System. Gene-specific primers for control and target genes were purchased from Applied Biosystems Incorporated (Table 3). The reagent kit used was the SuperScript III Platinum One-Step Quantitative RT-PCR System purchased from Invitrogen. Reverse transcription and amplification conditions were as follows: 53°C for 20-minutes, 95°C for 2-minutes, and 40 cycles at 95°C for 15 seconds and 60°C for 45 seconds. The cycle-threshold (Ct) data were imported into Microsoft Excel for normalization of target gene data to the control gene and for the calculation of fold changes in gene expression.

**Table 3:** Target sequences of the labelled probes used for detection of genes following amplification of cDNA using real time PCR

**Gene Name**	Gene Symbol*	Target Probe Sequence**
β-Actin	Actb	CTTCCTGGGTATGGAATCCTGTGGC

Hypoxanthine guanine phosphoribosyl transferase (HPRT)	Hprt	AGGGATTTGAATCATGTTTGTGTCA

Serine (or cysteine) peptidase inhibitor, claude E, member1 (PAI; plasminogen activator inhibitor-1)	Serpine1	CCTCATCCTGCCTAAGTTCTCTCTG

Coagulation Factor 3 (TF; Tissue Factor)	F3	AGAGTGTCCTGGGAGAAACACTCAT

Heme Oxygenase-1 (HO-1)	Hmox1	AAGGCTTTAAGCTGGTGATGGCCTC

Chemokine (C-X-C motif) ligand 2 (MIP-2; macrophage inflammatory protein-2; MIP-2)	Cxcl2	CCAAAAGATACTGAACAAAGGCAAG

Tumor Necrosis Factor alpha (TNF-α)	Tnf	TCACACTCAGATCATCTTCTCAAAA

Endothelin 1 (ET-1)	Edn1	GCTCCAGAAACAGCTGTCTTGGGAG

Endothelin Receptor A (ETA)	Ednra	GTGTTTAAGCTGTTGGCGGGGCGCT

Endothelin Receptor B (ETB)	Ednrb	GCCTACAAGCTGCTGGCAGGGGACT

*Gene symbols are as provided by the Applied Biosystems Incorporated.

**Target probe sequence represents the sequence which will hybridize with the PCR product and it is labeled such that once hybridized its fluorescence levels will be quantitatively detected by the ABI 7900 HT Sequence Detection System (Applied Biosystems). The forward and reverse primer sequences are proprietary and not provided by Applied Biosystems Inc.

### Cardiac histopathology

The whole heart of each non lavaged animal (n = 2) was submerged in fixative until processing for histology. Paraffin blocks were prepared from dehydrated tissues and 3- to 4-μm sections were stained with hematoxylin and eosin for light microscopic evaluation of the cardiac histopathology [9].

### Assessment of UfCPs-mediated systemic response

Haematological analysis, measurement of different biomarkers from plasma and serum were used for the assessment of systemic response following UfCPs exposure. Blood samples (blood A, Table 2) of each animal were collected from retro orbital sinus (haematology) and from abdominal aorta (biomarkers) on first and third day of recovery. For analysis of plasma renin concentration and activity, blood samples (blood B, Table 2) were also collected from caudal vein at several time points.

#### Haematology

For haematological analysis, 500 μl of blood sample from retro orbital sinus of each animal (blood A, n = 8) was collected in EDTA-Microvette and analysed by using haematology analyzer (Bayer ADVIA 120, Germany).

#### Acute phase proteins analysis

Blood samples collected from each animals (blood A, n = 8) were stored in aliquots of 2.6 ml in 2.9 ml *S-Monovette*^® ^tube (Sarstedt, Germany) with or without anticoagulant (citrate and EDTA) for further analysis of different markers. Each blood sample with anticoagulant was centrifuged (at 2710 g) for 10-minutes (4°C) for the collection of plasma sample and stored at -80°C until analysed. Fibrinogen concentration was measured from each plasma samples as previously described [60]. C-reactive protein (CRP) and haptoglobin (HP) were analysed from serum collected from blood samples by centrifugation for 15-minutes (at 1300 g, 4°C). CRP and HP were measured by using kit from DiaSorin Inc. (Stillwater, MN) for controls and standards, except the standard for CRP, which was obtained from Kamiya Biomedical Company (Seattle, WA).

#### Renin/Angiotensin analysis

Related to the limited amount of blood which can be taken repeatedly from a rat, blood samples collected from caudal vein (blood B, n = 8) before exposure, on the first, second, and third day of recovery were used to analyse only plasma renin concentration and activity. In this case 400 μl of blood samples from each animal were collected from the caudal vein in a 2 ml Eppendorf cup containing 25 μl mixture of 2.5 ml EDTA (Merck, Germany) and 25 μl of Phenantrolin (Calbiochem, Germany).

Furthermore concentration of angiotensin I (Ang I) and angiotensin II (Ang II) were determined from plasma on first and third recovery day. For this purpose blood samples were collected from abdominal aorta (blood A, n = 8) in a 5 ml falcon tube containing mixture (140 μl/ml blood) of p-hydroxy-mercuribenzoic acid (10 μl, Sigma), phenyl-methyl-sulfonyl-fluoride (10 μl, Sigma), EDTA (50 μl), pepstatin A (20 μl, Sigma) and o-phenanathrolin (50 μl, Merck). Measurements of renin activity and concentration as well as angiotensin (I and II) concentrations were assayed employing specific radioimmunoassays, as previously described [22].

### Statistics

After checking for the normal distribution assumption the differences between exposure and control groups were compared by using the t-test. Cardiovascular response parameters were described by a linear mixed regression model for repeated measurements. Based on this model group differences between the exposure and control group were tested. For expression analysis of various parameters from lung and heart tissues, a two-way analysis of variance (ANOVA) was used to analyze differences between the groups. However, for the plasma rennin and angiotensin (I and II) data the normailtiy assumption does not hold. Therefore, for plasma rennin and angiotensin (I and II) concentration the Wilcoxon rank sum test was performed. P values less than 0.05 were stated as statistically significant. All computations were done by the software packages Statgraphics plus v5.0 (Manugistics, Rockville, MD) and SAS V9.1 (Cary, NC). Data are presented as arithmetic mean values of n observations ± the standard error (SE), unless otherwise indicated.

## Abbreviations

Act: Physical activity; Ang I &II: Angiotensin I & II; BALF: Broncho-alveolar-lavage fluid; BP: Arterial blood pressure; CRP: C-reactive protein; ET-1: Endothelin-1; ETA: Endothelin A; ETB: Endothelin B; GGT: γ-Glutamyltransferase; HO-1: Hemeoxygenase-1; HP: Haptoglobin; HR: Heart rate; HRV: Heart rate variability; LF/HF: Low frequency (0.20 Hz to 0.75 Hz) to high frequency (0.75 Hz to 2.5 Hz) ratio; MIP-2: Macrophage inflammatory protein-2; NAG: N-acetyl glucosaminidase; UfCPs: ultrafine carbon particles; PAI-1: Plasminogen activator inhibitor-1; PRC: Plasma renin concentarton; RAS: Renin-angiotensin system; RMSSD: Square root of the mean of squared differences between adjacent NN (normal-to-normal) intervals; SE: Standard error; SDNN: Standard deviation of all normal sinus NN (normal-to-normal) intervals; SHR: Spontaneously hypertensive rats; T: Body core temperature; TF: Tissue factor; TNF-α: Tumor necrosis factor-alpha.

## Competing interests

The authors declare that they have no competing interests.

## Authors' contributions

SU, TS, and HS conceived and designed the experiments; SU, VH, RFT, MCS, MS-B, ST, EK, MB, AS, and UPK performed experiments; SU, PR and HS performed data analysis; SU, TS, UPK, and HS wrote the manuscript.
